# Comparison of Transcriptional Heterogeneity of Eight Genes between Batch *Desulfovibrio vulgaris* Biofilm and Planktonic Culture at a Single-Cell Level

**DOI:** 10.3389/fmicb.2016.00597

**Published:** 2016-04-27

**Authors:** Zhenhua Qi, Lei Chen, Weiwen Zhang

**Affiliations:** ^1^Laboratory of Synthetic Microbiology, School of Chemical Engineering & Technology, Tianjin UniversityTianjin, China; ^2^Key Laboratory of Systems Bioengineering (Ministry of Education), Tianjin UniversityTianjin, China; ^3^SynBio Research Platform, Collaborative Innovation Center of Chemical Science and EngineeringTianjin, China

**Keywords:** single-cell analysis, gene expression, mild steel, biofilm, planktonic, *D. vulgaris*

## Abstract

Sulfate-reducing bacteria (SRB) biofilm formed on metal surfaces can change the physicochemical properties of metals and cause metal corrosion. To enhance understanding of differential gene expression in *Desulfovibrio vulgaris* under planktonic and biofilm growth modes, a single-cell based RT-qPCR approach was applied to determine gene expression levels of 8 selected target genes in four sets of the 31 individual cells isolated from each growth condition (i.e., biofilm formed on a mild steel (SS) and planktonic cultures, exponential and stationary phases). The results showed obvious gene-expression heterogeneity for the target genes among *D. vulgaris* single cells of both biofilm and planktonic cultures. In addition, an increased gene-expression heterogeneity in the *D. vulgaris* biofilm when compared with the planktonic culture was also observed for seven out of eight selected genes at exponential phase, and six out of eight selected genes at stationary phase, respectively, which may be contributing to the increased complexity in terms of structures and morphology in the biofilm. Moreover, the results showed up-regulation of DVU0281 gene encoding exopolysaccharide biosynthesis protein, and down-regulation of genes involved in energy metabolism (i.e., DVU0434 and DVU0588), stress responses (i.e., DVU2410) and response regulator (i.e., DVU3062) in the *D. vulgaris* biofilm cells. Finally, the gene (DVU2571) involved in iron transportation was found down-regulated, and two genes (DVU1340 and DVU1397) involved in ferric uptake repressor and iron storage were up-regulated in *D. vulgaris* biofilm, suggesting their possible roles in maintaining normal metabolism of the *D. vulgaris* biofilm under environments of high concentration of iron. This study showed that the single-cell based analysis could be a useful approach in deciphering metabolism of microbial biofilms.

## Introduction

Propagation and metabolism of microorganisms on metal surfaces can change the physicochemical properties of these surface and cause the deterioration of metallic materials (Little et al., 1992; Thierry and Sand, 1995; Beech et al., 2000). Some representatives of microorganisms related to metal corrosion in both aerobic and anaerobic environments are the sulfate-reducing bacteria (SRB) (Voordouw, 1995; Hamilton, 2003; Enning and Garrelfs, 2014). The corrosive action of SRB is often associated with bacterial biofilms formed on the metal surfaces (Videla and Characklis, 1992; Dinh et al., 2004; Lewandowski and Beyenal, 2008). Although SRB biofilms have been extensively studied in the past decades and the previous studies have provided insights into SRB metal-reducing physiology and corrosion (Kolter and Losick, 1998; Liu et al., 2000; Fang et al., 2002; Hu, 2004), the differential gene expression between the biofilm and planktonic growth modes was less known until recent years. To accelerate understanding of the molecular mechanisms of SRB biofilm formation and maintenance, a whole-genome oligonucleotide microarray was used to examine differential expression patterns between planktonic populations and mature biofilm of *Desulfovibrio vulgaris* on a steel surface (Zhang et al., 2007). In addition, an integrated transcriptomic and proteomic analysis was recently conducted on mature *D. vulgaris* biofilm cells and compared to both batch and reactor planktonic populations (Clark et al., 2012). These results showed that the physiological differences between biofilm and planktonic cultures were caused by altered abundances of genes/proteins associated with carbon flow and extracellular structures; in addition, these studies have revealed the unique metabolic networks related to the formation and maintenance of *D. vulgaris* biofilm (Zhang et al., 2007; Clark et al., 2012). However, both these studies used population-averaged approach to describe biofilm behavior (Lazazzera, 2005), which did not take into consideration of potential differences (e.g., heterogeneous growth rates) between individual cells or different functional groups of cells in biofilms, and have resulted in possible biased conclusions (Beloin and Ghigo, 2005; An and Parsek, 2007; Stewart and Franklin, 2008; Hellweger and Bucci, 2009). To address this issue, alternative approaches that are able to capture differences between individual cells in micro-scale environments in biofilms need to be developed and evaluated (Lardon et al., 2011).

Recent studies have demonstrated that even homologous populations of microorganisms could have significant cell-to-cell gene expression heterogeneity (Lidstrom and Meldrum, 2003; Brehm-Stecher and Johnson, 2004; Strovas and Lidstrom, 2009; Stepanauskas, 2012; Blainey, 2013; Qi et al., 2014; Shi et al., 2014). In a previous study, the gene-expression levels of some selected genes of *D. vulgaris* were found to vary as much as 40 folds between cells of the same population, by using quantitative real-time reverse transcription-PCR (RT-qPCR) analysis (Qi et al., 2014). In the case of microbial biofilms, it is expected that such gene-expression heterogeneity between cells may be even more significant due to their obvious morphological, structural and even functional differences. Although works have been conducted in recent years using fluorescent reporter genes to visualize and measure microscale physiological heterogeneity in biofilms (Baty et al., 2000; Chai et al., 2008; Verplaetse et al., 2015), some limitations of the technique (i.e., requiring engineered strains; affecting the cell physiology by the energy required for expression of the reporter genes; and demanding oxygen for the activation of fluorescence) have restricted its application in biofilms (Stewart and Franklin, 2008). However, so far no single-cell based study has been conducted to analyze differential gene expression in *D. vulgaris* biofilm systems when compared with planktonic cells, and as thus potential gene-expression heterogeneity and its biological relevance in the *D. vulgaris* biofilm remains unclear.

In this study, with major aims to determine gene-expression heterogeneity between *D. vulgaris* cells grown in two different environments (i.e., biofilm and planktonic), and to further confirm the relationship between the selected genes and biofilm metabolism at a single-cell level, we applied a real-time reverse-transcription quantitative PCR (RT-qPCR) approach (Zhao et al., 2011; Shi et al., 2013; Qi et al., 2014). To do this, *D. vulgaris* biofilm was cultivated on mild steel (SS) slides to mimic microenvironments of metal corrosion. As an attempt to quantify heterogeneity levels of gene-expression between single cells in the *D. vulgaris* biofilm, the study could contribute to the further understanding of biofilm metabolism related to metal corrosion in natural environments.

## Materials and Methods

### Bacterial Strains and Growth Conditions

*Desulfovibrio vulgaris* subsp. *vulgaris* strain Hildenborough DSM 644 used in this study was obtained from the Deutsche Sammlung *von* Mikroorganismen und Zellkulturen (Braunschweig, Germany) and cultured in mineral medium as described by a previous publication (Zhang et al., 2007). Pure cultivation experiments were conducted in 200 mL serum bottles containing 70 mL of medium with lactate (38 mM) as the electron donor and sulfate (50 mM) as the electron acceptor according to previous publications (Zhang et al., 2006a; Qi et al., 2014). The *D. vulgaris* biofilm was formed on the surface of immersed SS slides in the same medium. SS slides (BST 503-2 SS slide, 0.7 cm × 5 cm and 1.2 mm thick) were purchased from BioSurface Technologies (Bozeman, USA). The SS slides were cleaned according to a previous study (Zhang et al., 2007) and then put into the medium to allow biofilm formation. The medium (with the immersed SS slides into it) was inoculated (5%) with a bacterial culture at middle-exponential phase (OD_595_ nm = 0.4), and all cultivation experiments were carried out at 35°C under anaerobic conditions, by using an anaerobic chamber (Fuma, Shanghai, China) for this purpose. These anaerobic conditions were achieved according to our previous publication (Qi et al., 2014). Planktonic cells were the non-attached floating cells under anaerobic cultivation conditions.

### Determination of Total Protein and Carbohydrate Levels of Biofilm and Planktonic Cells

Cells collected from the biofilm and planktonic cultures were re-suspended in 1 mL of 50 mM PBS (pH 6.5). For 1 mL cell suspension, 10 μL of phenylmethylsulfonyl fluoride (PMSF) was added. After cell suspensions were repeatedly frozen in liquid nitrogen and thawed at 25°C room temperature for three times, the suspended cells were broken by ultrasonication with an ultrasonic disrupter (Scientz, Ningbo, China) with ultrasonic amplitude transformer probe. The conditions for ultrasonic disruption was 5 s working time, 5 s interval time, 10 min total time under 60% ultrasonic power. Protein and carbohydrate levels were measured for the samples to confirm the growth status (biofilm vs. planktonic). The carbohydrate concentrations were measured using the anthrone-sulfuric acid colorimetry with sucrose as standard (Roe, 1954, 1955). Protein concentrations were determined with the Bradford assay and bovine serum albumin as the standard (Bradford, 1976) (**Supplementary Figure S1**).

### Selection of Target Genes for Single Cell RT-QPCR Analysis

Based on the functions of target genes and the relationships between genes and the *D. vulgaris* biofilm, 8 target genes (i.e., DVU0281, DVU0434, DVU0588, DVU1340, DVU1397, DVU2571, DVU2410 and DVU3062) were selected for the following single-cell analysis (Supplementary Table S1). The functions of the 8 target genes and 3 candidate internal control genes in the *D. vulgaris* biofilm have been examined in previous studies, and the results are presented in **Table 1**. The gene expression levels of DVU0588, DVU0434, and DVU2410 under biofilm-growth conditions were down-regulated when compared to its planktonic culture (Jenney et al., 1999; Zhang et al., 2007); while their expression levels in the biofilm on the glass slides were up-regulated in comparison with planktonic culture in another transcriptomic and proteomic analysis (Clark et al., 2012). In addition, it was proposed that DVU0281 might be related to the formation and metabolism of the *D. vulgaris* biofilm (Ren et al., 2004). To investigate the role of iron in *D. vulgaris* biofilm formation process, DVU2571, DVU1340, and DVU1397 were selected in this study. In addition, a previous study showed that expression level of several two-component signal systems was differentially regulated in the *D. vulgaris* biofilm, suggesting their possible roles related to biofilm formation and maintenance (Zhang et al., 2007). Among the results, DVU3062 was selected, as previous studies suggested that most of hybrid-type histidine kinases found in bacteria could be involved in signal transduction required for cell-cell communication or differentiation (Slater et al., 2000; Takeda et al., 2001; Zhang et al., 2006b).

**Table 1:** Selected genes and optimized primers for single-cell analysis.

Gene ID⋆	Gene name	Annotation	Primer names	Sequence (5′– > 3′)	Functions of the genes in *Desulfovibrio vulgaris* biofilm
DVU0281		Exopolysaccharide biosynthesis Protein	DVU0281A-fw DVU0281A-rv	TACCCCTGATTCTACCCGTCA GGTGCGAAATTT GAGGATGTC	Biofilm formation and metabolism (Ren et al., 2004)
DVU0434		Ech hydrogenase Subunit EchA	DVU0434A-fw DVU0434A-rv	CCTCGGCTACATGAAAGAGCAC AGCGTGGTGATTTCCCAGAAG	Energy conversion and carbon flow (Jenney et al., 1999; Zhang et al., 2007; Clark et al., 2012)
DVU0588		Formate dehydrogenase subunit beta	DVU0588C-fw DVU0588C-rv	ATGAACTTCGGCGATGAGCAG CGCATGTTCGTAGAAGTCCTTG	
DVU1340		Fur family transcriptional regulator	DVU1340B-fw DVU1340B-rv	AAGTCCGCTTCGACGGCAT ACCGGATTGGCTGTCCGAAC	
DVU1397	bfr	Bacterioferritin	DVU1397D-fw DVU1397D-rv	GCGAAAGTCATCGAAGTGCTGA CGGCAAGTTCTCCGTAGTCCAT	Biofilm formation and maintenance (Zhang et al., 2007; Clark et al., 2012)
DVU2571	*feoB*	Ferrous iron	DVU2571D-fw	GTCGCGAGAAGCTTGCAACACT	
		Transport protein	DVU2571D-rv	AAGAAGATGCCGACGATGAGGA	
		B			
DVU2410	*sodB*	Superoxide dismutase	DVU2410B-fw DVU2410B-rv	CCATGAGACTCGAAGACGTGGT TCATGCCTGCCCAGTAGAAGGT	Reactive oxygen species or general stress resistance (Clark et al., 2012)
DVU3062		Sensor histidine kinase/response regulator	DVU3062D-fw DVU3062D-rv	TTGGCGTGCAGGTGAATGA CGTTCAAGCGTTGCAAATCC	Signal transduction (Slater et al., 2000; Takeda et al., 2001; Zhang et al., 2006b)
DVU0565	*gap-1*	Glyceraldehyde	DVU0565D-fw	TATGACCCGCAGAAGCACCAT	Housekeeping gene
		3-phosphate dehydrogenase	DVU0565D-rv	CGATGCCGTACTTCTCTTGGAT	(Marco and Kleerebezem, 2008; Zhao et al., 2011)
DVU1090	*recA*	Recombinase A	DVU1090H-fw DVU1090H-rv	GCCGTCATCTTCATCAACCAG TCCATACGGACGGAACTGTAGA	Housekeeping gene (Qi et al., 2014)
Dvl6SA	*rrsA*	16S ribosomal	Dvl6SAC-fw Dvl6SAC-rv	CAACCCCTATTGCCAGTTGCT GCCATGATGACTTGACGTCGT	Housekeeping gene (Scholten et al., 2007; Plugge et al., 2010)

*⋆IDs of the genes are from Kyoto Encyclopedia of Genes and Genomes.*

### Selection and Evaluation of Primers

Several previous single-cell studies have found that RT-PCR primers functioning well for bulk cells may not be successfully applied to single-cell analysis (Zhao et al., 2011; Shi et al., 2013; Qi et al., 2014), so that a primer has to be validated at a single-cell level. Typically, for a given target gene, one pair of single-cell primers were screened and validated from 4 to 9 pairs of candidate primers functional well at a bulk-cell level (Supplementary Table S1). The criteria and method for screening and validation of single-cell primers were the same as previously described (Zhao et al., 2011; Shi et al., 2013; Qi et al., 2014). For each single cell, three analytical replicates were analyzed (Shi et al., 2013; Qi et al., 2014).

### Selection of Internal Control Genes

To minimize differences between single cells due to sample preparation (i.e., RNA yield, quality and efficiency of the reverse transcription), it was supposed to normalize the resulting threshold cycle (C_T_) data obtained from RT-qPCR analyses against an internal control gene (Heid et al., 1996; Ståhlberg et al., 2013). So far little was known about the degree of expression stability of these internal control genes in the *D. vulgaris* biofilm cells. In addition, our previous study showed that cell-to-cell expression levels of several internal control genes widely used for bulk-cell studies, such as DVU1090 encoding recombinase A, DVU0600 encoding *L*-lactate dehydrogenase, and Dv16SA encoding 16S ribosomal RNA, were different among the single cells from both *D. vulgaris* monoculture and coculture (Qi et al., 2014). To obtain a suitable internal control gene for the purpose of comparing gene-expression heterogeneity of the *D. vulgaris* grown in biofilm and planktonic cultures, several potential candidates as internal control genes were evaluated at the single-cell level. According to previous studies about *D. vulgaris* and other similar species at the bulk-cell level, we selected three housekeeping genes as potential internal control genes for subsequent single-cell analysis, i.e., 16S ribosomal RNA gene (Dv16SA) (Scholten et al., 2007; Plugge et al., 2010), glyceraldehyde 3-phosphate dehydrogenase gene (DVU0565, *gap-1*), and recombinase A gene (DVU1090, *recA*) (Marco and Kleerebezem, 2008; Zhao et al., 2011). Toward this goal, we randomly isolated two groups of *D. vulgaris* single cells (each containing 12 single cells) from *D. vulgaris* biofilm and planktonic culture, respectively, conducted RT-qPCR analysis of three internal control genes for all single cells, and then calculated the mean and standard deviations (SDs) of C_T_ values from single-cell RT-qPCR using the OriginPro 8.0 software.

### Planktonic and Biofilm Cells Collection Procedure

Planktonic cells from the surrounding medium were transferred to centrifuge tubes with O-ring seals under anaerobic conditions in the anaerobic chamber and collected by centrifuging at room temperature (6,000 ×*g*). To keep their gene expression profiles intact, the planktonic cells were put into a RNALater solution immediately (Ambion, Carlsbad, CA, USA) (Bachoon et al., 2001; Mason et al., 2012; Shi et al., 2013; Qi et al., 2014).

For the *D. vulgaris* biofilms, superficial cells of the mature biofilm formed on the surface of SS slides were slightly washed off with 50 mM oxygen-free phosphate-buffered saline (PBS, pH 6.5) using a dropper under anaerobic conditions in the anaerobic chamber, then inner mature biofilm cells were scraped using a sterile razor blade from the surface of SS slides. To keep their gene expression profiles intact, the biofilm cells were also put into a RNALater solution immediately (Ambion, Carlsbad, CA, USA) (Zhang et al., 2007). The suspension of biofilm cells was then heavily vortexed to release single *D. vulgaris* cells to be used for single-cell isolation (Chalmers et al., 2007; Marcy et al., 2007; Zhang et al., 2007).

### Single-Cell Isolation, RNA Extraction, cDNA Synthesis and Real-Time Quantitative PCR

Single cells were isolated using an inverted IX71 microscope connected with a single cell manipulator (Olympus Inc, Japan) from the cell suspension according to previous publications (Shi et al., 2013; Qi et al., 2014). Total RNAs were extracted from the single cells by a ZR RNA MicroPrep kit (Zymo Research, Irvine, CA, USA) and then used for synthesize cDNAs by a SuperScript VILO MasterMix (Invitrogen, Carlsbad, CA, USA). Finally, the cDNAs were used as templates for quantitative PCR analysis by using Power SYBR Green PCR master mix (Invitrogen, Carlsbad, CA, USA) on an ABI StepOne real-time PCR system (Applied Biosystems, Foster, CA, USA) as previously described (Qi et al., 2014).

In this study, approximately 31 single *D. vulgaris* cells were randomly isolated from three culture bottles under the identical growth and subjected to the single-cell RT-qPCR analysis from each biological sample of the biofilm and the planktonic cultures. To ensure no significant change of gene expression profiles, the sample cells were put into a RNALater solution and the single-cell isolation process for 31 cells was completed within 30 min (Bachoon et al., 2001; Mutter et al., 2004; Uhlenhaut and Kracht, 2005; Dekairelle et al., 2007).

### Data Analysis

To ensure data reproducibility, all growth experiments and measurements were repeated three times. For single-cell based RT-qPCR analysis, we performed the ^Δ^C_T_ relative quantification method with DVU1090 (recombinase A) as the internal reference to normalize the resulting threshold cycle (C_T_) data. Non-parametric statistic tests were used to analyze the distribution variation of gene expression levels of single cells (Siegel, 1957). In addition, the Mann–Whitney, Kruskal–Wallis, and Kolmogorov-Smirnov analysis of variance (ANOVA) tests were also utilized to analyze the relationship between four different groups of RT-qPCR measurements (i.e., biofilm and planktonic cultures, exponential and stationary phases) using the OriginPro 8.0 software (OriginLab Corporation, Northampton, MA, USA). Correspondence analysis (CA) was conducted using the IBM SPSS Statistics 20 software (IBM, Chicago, IL, USA) to reveal differences between the various categories in the same variable (genes or growth conditions) and to determine relationships and associations between genes and growth modes (Greenacre, 1984).

## Results And Discussion

### *D. vulgaris* Biofilm Formation on Mild Steel Surface and Determination of Total Protein and Carbohydrate Levels

**FIGURE 1:**
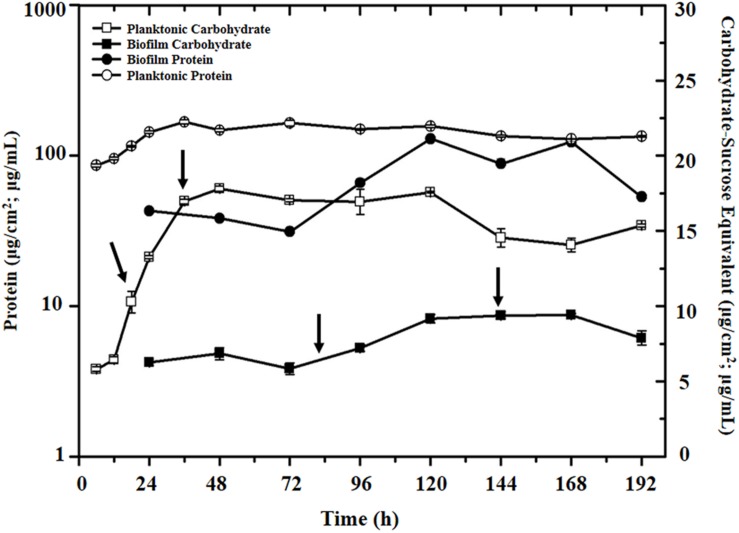
**Protein and carbohydrate levels in the biofilm and planktonic cells during the growth period.** The arrows in the plot indicated the four sampling time points for single-cell RT-qPCR analysis (i.e., 18 and 36 h for planktonic culture, and 84 and 144 h for biofilm, respectively).

To investigate gene-expression levels of selected target genes potentially associated with SRB biofilm formation and metal corrosion, *D. vulgaris* biofilm was formed on the immersed SS slides in 200 mL serum bottles with lactate (38 mM) as electron donor and sulfate (50 mM) as electron acceptor. Typically, when *D. vulgaris* cells was cultured at 35°C for 6 days, the SS slides were completely covered by *D. vulgaris* biofilm with average 1-2 mm in thickness (**Supplementary Figure S2**). To monitor growth status of *D. vulgaris* in both biofilm and planktonic cultures and determine sampling points for further single-cell based analysis, both protein and carbohydrate levels in the biofilm and planktonic cells were measured as previously described (Clark et al., 2012) (**Figure 1**). The results showed that protein and carbohydrate levels approached steady-state stages at 36 h for planktonic culture and at 144 h for biofilm, respectively (**Figure 1**). At the steady-state stages, the planktonic cultures had protein concentration of 167 ± 5.2 μg/mL, carbohydrate concentration of 15.9 ± 0.6 μg/mL and a carbohydrate: protein ratio (C:P) of approximately 0.10 (μg/μg), while the biofilm had a protein level of 88 ± 7.0 μg/cm^2^ and a carbohydrate level of 9.7 ± 0.2 μg/cm^2^ with an approximately C:P ratio of 0.11 (μg/μg), consistent with a previous conclusion that *D. vulgaris* did not produce a carbohydrate-rich biofilm matrix (Clark et al., 2007).

To compare gene-expression heterogeneity between biofilm and planktonic cultures, *D. vulgaris* cells at middle-exponential and stationary phases from both biofilm and planktonic cultures were collected (i.e., 18 and 36 h for planktonic culture, and 84 and 144 h for biofilm, respectively) (**Figure 1**). To minimize variation during the sampling process, the total sampling time was completed within 2 h from the collection of the samples to the synthesis of the cDNA to keep their gene expression profiles intact (Qi et al., 2014). The cDNAs were stored at –20°C until all the cDNAs at 4 time periods were synthesized, and used for qPCR analysis.

### Selection of Target Genes and Optimization of Primers for Single Cell RT-QPCR Analysis

To compare gene-expression heterogeneity between biofilm and planktonic cultures of *D. vulgaris*, 8 target genes (i.e., DVU0281, DVU0434, DVU0588, DVU1340, DVU1397, DVU2571, DVU2410, and DVU3062) potentially related to formation and metabolism of the *D. vulgaris* biofilm and three candidate genes of internal reference (i.e., DVU0565, DVU1090, and Dv16SA) were selected (Supplementary Table S1). To improve the success rate of the following single-cell analysis, primers of the eight target genes and three candidate internal reference genes has to be verified at a single-cell level. In this study, we evaluated 49 pairs of primers (a total of 17 pairs for three internal control genes and a total of 32 pairs for eight selected genes) at the single-cell level (Supplementary Table S1). The validation results of single-cell primers for each target gene were listed in **Table 1**.

### Selection and Evaluation of Internal Control Genes

Based on previous studies, three candidate internal control genes (i.e., DVU0565, DVU1090, and Dv16SA) were selected for the following single-cell analysis (Supplementary Table S1). To obtain an optimal reference gene for the single-cell analysis, we evaluated the three candidate internal control genes. The results showed that the SD were 0.74, 1.23, and 0.88 cycles across 12 single cells from the planktonic culture, and 0.83, 2.03, and 1.26 cycles across 12 single cells from the mature biofilm, for internal control gene DVU1090, Dv16SA and DVU0565 genes, respectively (**Supplementary Figure S3**). Compared with the other two potential internal control genes, the SD of DVU1090 (*recA*) were lower among individual cells from both biofilm and planktonic cultures (Takle et al., 2007; Marco and Kleerebezem, 2008), so that DVU1090 was selected as the internal control gene for the subsequent single-cell analysis. For Dv16SA gene that was often preferentially selected as an internal control in bulk-cell based studies (Bustin et al., 2009; Plugge et al., 2010), our results showed that it carried a significant gene-expression heterogeneity across single cells, so it may not be optimal as an internal control gene for gene expression analysis at the single-cell level. In addition, the analysis showed that gene-expression heterogeneity of all three internal control genes was greater in the *D. vulgaris* single cells from biofilm than those from planktonic culture, in agreement with the observation that the biofilm tended to have a higher degree of heterogeneity and complexity as compared with planktonic culture (Stewart and Franklin, 2008).

### Reliability Analysis of Single-Cell Gene Expression Data

In this study, one internal control gene (DVU1090) and eight selected target genes (i.e., DVU0281, DVU0434, DVU0588, DVU1340, DVU1397, DVU2410, DVU2571, and DVU3062) were analyzed in each of the 31 individual cells isolated from each growth condition (i.e., biofilm and planktonic cultures, exponential and stationary phases). A quality control was manually conducted for a total 3,348 reactions (i.e., 31 cells × 4 conditions × 9 genes × 3 analytical replicates). As a quality control for reliable data, poor RT-qPCR reactions, i.e., wrong amplification peaks in the melting curves; large variations (i.e., SD > 0.5) among analytical triplicates, were removed, resulting in a total of 29, 30, 28, and 28 cells out of 31 individual cells with all nine genes successfully analyzed at exponential phase in biofilm and planktonic cultures, and at stationary phase in biofilm and planktonic cultures, respectively. The amplification efficiency of all RT-qPCR data was greater than 93%. The results indicated that variations among technical replicates of RT-qPCR analysis were small for all eight genes, suggestive of good reproducibility of the data (Supplementary Table S2).

### Gene-Expression Heterogeneity in *D. Vulgaris* Cells from Biofilm and Planktonic Culture

**FIGURE 2:**
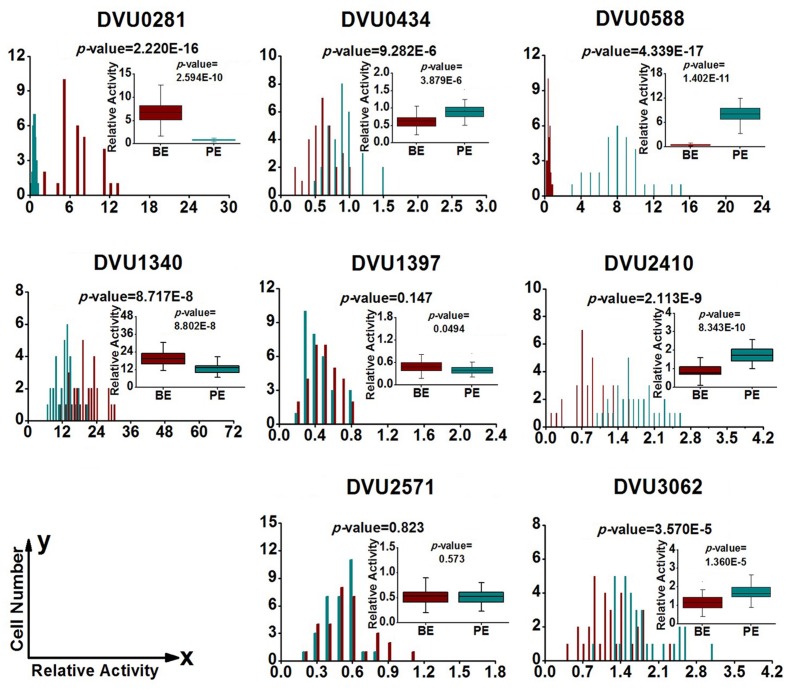
**Gene-expression distribution of target genes at exponential phase in the biofilm and planktonic cultures.** BE (Dark Red) and PE (Dark Cyan) are abbreviations for exponential phase in the biofilm and the planktonic culture, respectively. *P*-values in the histograms were determined by using the non-parametric two-sample Kolmogorov–Smirnov test between the biofilm and planktonic cultures (α = 0.05). The *x* axis shows the relative activity of a specific gene compared with the internal control gene DVU1090 in the same cell, and the *y* axis shows the number of cells that have the same relative activity. Box plots of single-cell gene expression levels and *P-*values associated with the differences between biofilm and planktonic culture. *P-*values in the box plots were calculated by means of the two-tailed non-parametric Mann–Whitney statistical significance test between biofilm and planktonic culture. Box plots show following statistical values: Open square – mean, solid line – median, upper and lower box lines – the 75th and 25th percentiles, respectively. Top and bottom horizontal solid line – maximal and minimal values, respectively.

**FIGURE 3:**
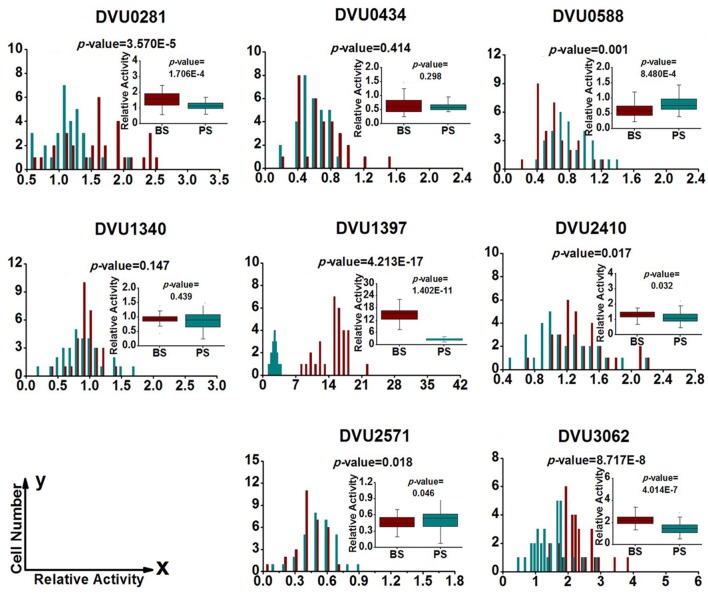
**Gene-expression distribution of target genes at stationary phase in the biofilm and planktonic cultures.** BS (Dark Red) and PS (Dark Cyan) are abbreviations for stationary phase in the biofilm and the planktonic culture, respectively. For statistical analysis, please refer to the figure legend of **Figure 2**.

**FIGURE 4:**
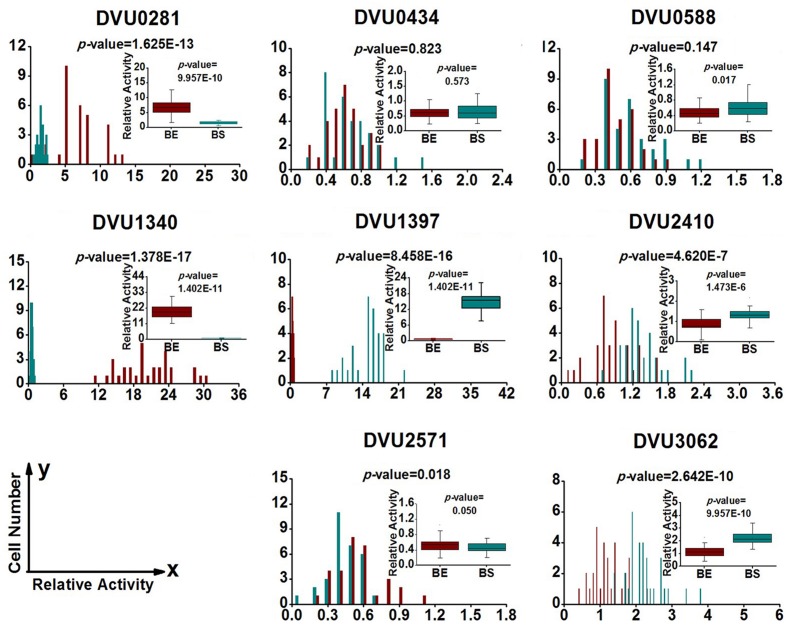
**Gene expression distribution of target genes at exponential and stationary phases in the biofilm.** BE (Dark Red) and BS (Dark Cyan) are abbreviations for exponential phase and stationary phase in biofilm, respectively. For statistical analysis, please refer to the figure legend of **Figure 2**.

**FIGURE 5:**
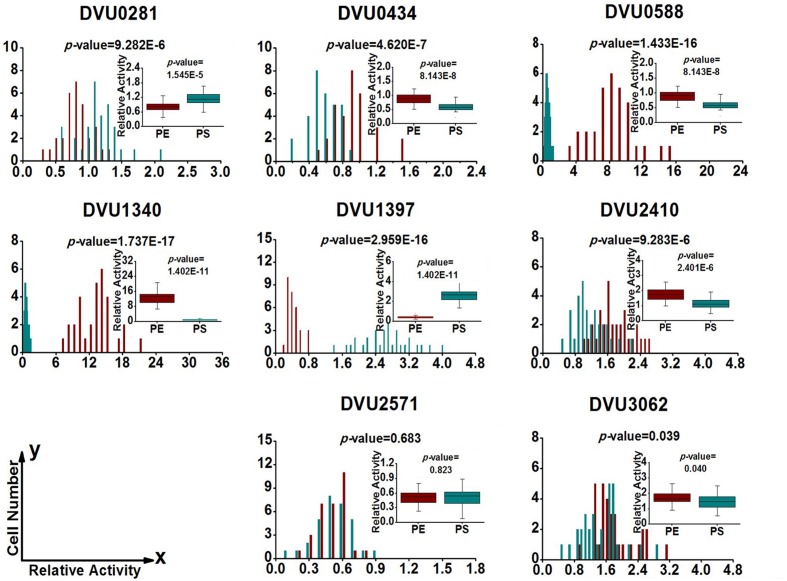
**Gene expression distribution of target genes at exponential and stationary phases in planktonic culture.** PE (Dark Red) and PS (Dark Cyan) are abbreviations for exponential phase and stationary phase in planktonic culture, respectively. For statistical analysis, please refer to the figure legend of **Figure 2**.

To quantify heterogeneity existed in the *D. vulgaris* cultures; we drew distribution histograms based on relative activity of all eight target genes under four growth conditions. The relative activity data for all single cells under four conditions was provided in Supplementary Table S3. In addition, to conveniently compare biofilm with planktonic culture, we made four combinations of distribution histograms for four growth conditions (i.e., planktonic *vs*. biofilm at exponential phase, planktonic *vs*. biofilm at stationary phase, exponential *vs*. stationary in planktonic culture, and exponential *vs*. stationary in biofilm) (**Figures 2–5**). Similarity between gene-expression distributions was evaluated using *p*-values determined by non-parametric two-sample Kolmogorov–Smirnov tests. In addition, in the box plots, similarity of gene expression between conditions was evaluated using *p*-values calculated by two-tailed non-parametric Mann–Whitney statistical significance tests. To more accurately describe the heterogeneity levels, we first defined degree of gene-expression heterogeneity as the fold between the highest and the lowest gene expression levels for each target gene in all single cells under each growth condition, and data was presented in **Table 2** accordingly. The results showed that the distribution span of relative gene-expression levels along the horizontal axis reached up to 15, e.g., DVU1397 at stationary phase in the biofilm (**Figure 3**), suggesting that all target genes carried significant gene-expression heterogeneity in all four conditions. For instance, the differences in terms of relative gene-expression levels could be greater than approximately 80 folds between single cells for DVU0281 in the biofilm at exponential phase, and approximately 50 folds between single cells for DVU3062 at stationary phase in the planktonic culture (**Table 2**). These results demonstrated that the gene-expression heterogeneity might be a common phenomenon in both biofilm and planktonic cultures. In addition, among all eight genes, the gene-expression heterogeneity for DVU0281 encoding exopolysaccharide biosynthesis protein tended to be the largest among single cells under exponential phase in *D. vulgaris* biofilm, while DVU2571 involved in ferrous iron transport tended to be the largest under stationary phase in *D. vulgaris* biofilm (**Table 2**). Although it is still unknown the specific relationship between the heterogeneity and functionality and physiology of biofilms, high level of heterogeneity for various cellular components is commonly found in biofilm. For example, studies have demonstrated that the distribution of exopolysaccharides in biofilms is highly uneven, which may be related to the formation of the three-dimensional biofilm structures (Laue et al., 2006; Stewart and Franklin, 2008). In addition, the non-uniform multilevel exopolysaccharide matrix is likely one of the main reasons for the stability of biofilms (Jefferson, 2004). Therefore, the highest heterogeneity of DVU0281 (involved in exopolysaccharide biosynthesis) in terms of gene expression might be vital for the *D. vulgaris* biofilm. Furthermore, according to the previous studies, mature biofilms contain concentration gradients of metabolic substrates and products, including metal ions (Stewart and Franklin, 2008; Chen et al., 2011). Although iron plays significant roles in biofilm formation in many microorganisms, several studies have indicated that excess iron was detrimental to biofilms (Banin et al., 2005; Hindré et al., 2008). Therefore, to maintain appropriate iron concentration in different structure levels of the *D. vulgaris* biofilm, higher gene-expression heterogeneity of DVU2571 involved in ferrous iron transport may be in agreement with the above observation, especially for *D. vulgaris* biofilm growth on the SS. By comparing the gene-expression heterogeneity of each target gene under the four growth conditions, the results showed that DVU1340 encoding a Fur regulator and DVU3062 encoding sensor histidine kinase/response regulator were the most heterogeneously expressed at stationary phase in planktonic culture (**Table 2**). The lower heterogeneity levels of regulatory genes in biofilm might be important in maintaining delicate regulation of the genes involved in biofilm formation and maintenance, as proper regulation and assembly of the matrix components are key determinant to the biofilm formation (Kearns, 2008).

**Table 2:** Maximum gene-expression heterogeneity among 31 single-cells for eight target genes under four growth conditions.

Growth Conditions	DVU0281		DVU0434		DVU0588		DVU1340		DVU1397		DVU2410		DVU2571		DVU3062	
	Max-H☆	Mean-H ± SD⋆	Max-H	Mean-H ± SD	Mean-H	Max-H ± SD	Max-H	Mean-H ± SD	Max-H	Mean-H ± SD	Mean-H	Max-H ± SD	Max-H	Mean-H ± SD	Mean-H	Max-H ± SD
BE	80.312	4.571 ± 11.503	4.607	1.626 ± 0.644	14.266	1.965 ± 0.162	2.652	1.362 ± 0.301	4.452	1.613 ± 0.597	20.704	2.460 ± 2.825	5.400	1.634 ± 0.635	5.746	1.642 ± 0.649
PE	4.312	1.461 ± 0.529	3.004	1.343 ± 0.312	4.526	1.528 ± 0.531	3.102	1.38 ± 0.337	3.789	1.548 ± 0.497	2.608	1.366 ± 0.303	3.522	1.445 ± 0.410	3.441	1.386 ± 0.358
BS	4.240	1.572 ± 0.591	5.989	1.685 ± 0.665	5.149	1.607 ± 0.576	3.171	1.299 ± 0.332	2.946	1.308 ± 0.313	5.198	1.431 ± 0.333	13.573	1.853 ± 1.948	2.761	1.312 ± 0.283
PS	3.445	1.406 ± 0.390	4.394	1.499 ± 0.853	3.603	1.475 ± 0.412	7.110	1.676 ± 0.784	2.919	1.338 ± 0.308	4.651	1.525 ± 0.501	11.306	1.820 ± 1.469	50.071	3.095 ± 6.444

*☆Max-H is abbreviation of maximum gene-expression heterogeneity; The gene-expression heterogeneity values were calculated by dividing the highest gene expression levels by the lowest gene expression levels.*

*⋆Mean gene-expression heterogeneity ± Standard Deviation. BE and PE are abbreviations of exponential phase for biofilm and planktonic cells, respectively. BS and PS are abbreviations of stationary phase for biofilm and planktonic cells, respectively.*

By comparing the gene-expression distributions of the eight target genes, the results showed that gene-expression levels under certain growth conditions were obviously distinct for one certain target gene between different biofilm cells, suggesting significant stochastic gene expression in the *D. vulgaris* biofilm (**Figures 2–5**). Although chemical heterogeneity and physiological adaptation to the local environment explain much of the biological heterogeneity in biofilms, it is likely that stochastic gene expression also contributes to the phenotypic heterogeneity (Stewart and Franklin, 2008). Researchers have noted obvious variation in terms of activity of single bacterial cells in biofilms (Davies and Geesey, 1995; Baty et al., 2000). It has been proposed that stochastic gene expression can also commendably explain phenotypic heterogeneity in a biofilm population that is independent of the prevailing environmental conditions (Chai et al., 2008; Stewart and Franklin, 2008). With the increased complexity in biofilms, we hypothesized that increased gene-expression heterogeneity may also occur in the *D. vulgaris* biofilms when compared with the planktonic culture since the gene-expression heterogeneity could lead to different fates for individual cells (Colman-Lerner et al., 2005; Strovas et al., 2007). To validate this hypothesis, we also calculated the SD for each gene among the single cells as a second indicator. In general, similar trends were observed using both indicators (**Table 2**). By comparing gene-expression heterogeneity of all eight genes under the four growth conditions, the results indicated that gene-expression heterogeneity of six genes (i.e., DVU0281, DVU0434, DVU0588, DVU1397, DVU2410, and DVU2571) were increased at both exponential and stationary phases for the biofilm when compared with the planktonic culture, and gene-expression heterogeneity of DVU3062 was increased slightly at exponential phase while decreased significantly at stationary phase for the biofilm (**Table 2**). The only exception was DVU1340 whose gene-expression heterogeneity was decreased at both exponential and stationary phases in the *D. vulgaris* biofilm (**Table 2**). In addition, the results showed decreased heterogeneity levels for two regulatory genes (i.e., DVU1347 and DVU30162) at stationary phase in biofilm, which may worth further investigation for possible mechanism. Overall, compared with the planktonic culture, gene-expression heterogeneity of target genes was increased at both exponential and stationary phases for the biofilm. To demonstrate that the gene-expression heterogeneity resulted from the growth states of biofilm instead of the variances among biological replications, we compared the gene-express histograms and heterogeneity boxplots (**Supplementary Figure S4**) and the heterogeneity frequency distribution histograms (**Supplementary Figure S5**) for target gene DVU0281 that encodes a exopolysaccharide biosynthesis protein, relevant to biofilm formation) from biological replicates in both planktonic and biofilm cultures. The results showed that the mean gene-expression heterogeneity levels of three biological replicates for the biofilm were about the same (i.e., 7.931, 8.332, and 7.537); similarly, the mean gene-expression heterogeneity levels of three biological replicates for planktonic culture were also about the same (i.e., 1.622, 1.580, and 1.755). Meanwhile, the mean heterogeneity levels between biofilm and planktonic cultures were more than five times different for DVU0281 under the exponential phase, suggesting that different heterogeneity levels were resulted from different growth state (biofilm *vs*. planktonic) rather than from variation between biological replicates. In addition, based on the studies on aerobic and facultative bacteria, the phenotypic heterogeneity and complexity within biofilm were greater when compared with the planktonic culture (Stewart and Franklin, 2008). Therefore, although further analysis of more genes is still needed, it is speculated that a possible linkage between increased gene-expression heterogeneity and phenotypic heterogeneity and structural complexity in the *D. vulgaris* biofilm compared to the planktonic culture.

### Regulation of Target Genes in *D. Vulgaris* Cells from Biofilm and Planktonic Culture

To determine the differentially regulated genes between the different growth conditions using single-cell datasets, we established four pairs of comparisons for eight target genes (**Figures 2–5**), with an emphasis to the first two pairs of comparisons that may reveal genes functionally closely related to the biofilm metabolism. The comparison of planktonic culture *vs*. biofilm is presented in **Figure 2** for the exponential-phase cells, while **Figure 3** presents the same comparison for the stationary-phase cells. The results showed that: (i) the distributions of three genes (DVU0281, DVU1340, and DVU1397) were clearly shifted toward right side in the plots at both growth phases in the biofilm, representing possible up-regulation of gene expression in single cells of the *D. vulgaris* biofilm, suggesting that these genes may be related to formation and metabolism of biofilm (**Figures 2** and **3**); (ii) both *p*-values of the distribution and box plots analysis of DVU1340 were greater than 0.05 at stationary phase (**Figure 3**), suggesting that DVU1340 may be functional primarily at fast-growing exponential phase; (iii) the distributions of three target genes, DVU0434, DVU0588, and DVU2571 were shifted toward left side at both growth phases in the biofilm with compared to the planktonic culture, suggesting that the genes were down-regulated and they may not be directly related to the formation and metabolism of biofilm (**Figures 2** and **3**); (iv) DVU2410 and DVU3062 were down-regulated at exponential phase in the biofilm but up-regulated at stationary phase in the biofilm (**Figures 2** and **3**), suggesting the two genes may be related to late-stage function of mature biofilm, such as maintenance, although further proof is still needed.

It has been found that excess iron could be detrimental to biofilms (Johnson et al., 2005; Hindré et al., 2008). In this study, the *D. vulgaris* biofilm was formed on SS slides, which may lead to a high concentration of iron around the biofilm. Our analysis showed that DVU1340 encoding ferric uptake repressor (Fur) family transcriptional regulators and DVU1397 encoding ion storage protein bacterioferritin were up-regulated, while DVU2571 encoding a ferrous iron transport protein was down-regulated in the *D. vulgaris* biofilm (**Table 3**), suggesting that these genes may be important in maintaining the normal metabolism of the *D. vulgaris* biofilm under a high concentration of iron, although further investigation is still needed to reveal the related mechanism.

**Table 3:** The average relative activity among 31 single-cells for eight target genes under four growth conditions.

Growth conditions	DVU0281	DVU0434	DVU0588	DVU1340	DVU1397	DVU2410	DVU2571	DVU3062
BE	6.848 ± 3.027⋆	0.617 ± 0.210	0.461 ± 0.172	19.987 ± 4.898	0.494 ± 0.167	0.850 ± 0.364	0.553 ± 0.204	1.190 ± 0.432
PE	0.808 ± 0.218	0.924 ± 0.229	8.196 ± 2.616	12.878 ± 3.231	0.432 ± 0.159	1.758 ± 0.427	0.506 ± 0.137	1.779 ± 0.495
BS	1.605 ± 0.504	0.678 ± 0.276	0.601 ± 0.224	0.934 ± 0.190	14.904 ± 3.070	1.367 ± 0.340	0.446 ± 0.134	2.238 ± 0.523
PS	1.152 ± 0.308	0.590 ± 0.167	0.812 ± 0.248	0.901 ± 0.328	2.646 ± 0.596	1.179 ± 0.386	0.520 ± 0.173	1.468 ± 0.547

*BE and PE are abbreviations of exponential phase for biofilm and planktonic cells, respectively. BS and PS are abbreviations of stationary phase for biofilm and planktonic cells, respectively. ⋆Average Relative Activity±Standard Deviation; The relative activity are calculated by using ^Δ^CT relative quantification method with DVU1090 as the internal reference to normalize the resulting threshold cycle (CT) data.*

To determine the functioning periods of the genes in biofilm and planktonic cultures, the gene-expression distribution was also compared between exponential and stationary phases (**Figures 4** and **5**). The results showed that the distribution patterns of 4 out of the 8 genes (i.e., DVU0281, DVU0588, DVU1340, and DVU2571) were shifted toward right at the exponential phase in the biofilm (**Figure 4**), with statistical significances of both non-parametric two-sample Mann–Whitney and Kolmogorov–Smirnov tests less than 0.05, suggesting that they might be functional primarily in exponential phase in the *D. vulgaris* biofilm. Meanwhile, the distribution patterns of three genes (DVU1397, DVU2410, and DVU3062) shifted toward left at exponential phase in the biofilm with the *p*-values less than 0.05 (**Figure 4**).

### Correspondence Analysis of Single-Cell Data

**FIGURE 6:**
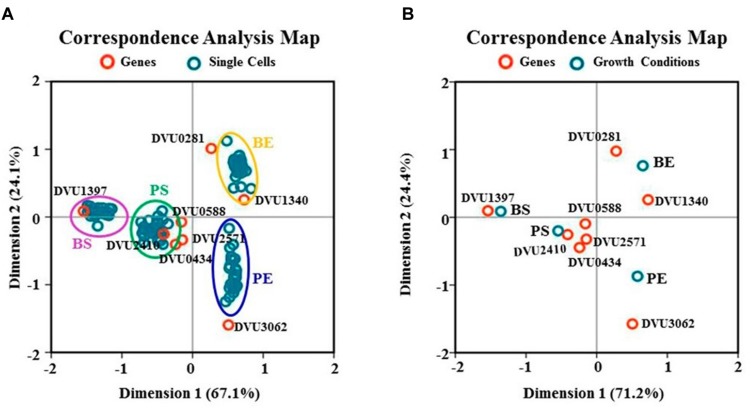
**Correspondence analysis.** **(A)** CA plot of eight target genes and 124 individual cells under four growth conditions; **(B)** CA plots of eight target genes and four growth conditions. BE and PE are abbreviations for exponential phase in the biofilm and the planktonic culture, respectively. BS and PS are abbreviations for stationary phase in the biofilm and the planktonic culture, respectively.

A statistical analysis technique of multivariate dependent-variables, namely CA, was also applied to the single-cell datasets (Kishino and Waddell, 2000). Two CA plots were generated independently; one was based on the relationship between target genes and all single cells of four growth conditions (**Figure 6A**), and another was based on the relationship between target genes and four growth conditions where the conditions were represented by the C_T_ means of all 31 single cells (**Figure 6B**). The CA analysis showed that a cumulative inertia (that represents the explanation levels of each dimension to the differences between the various categories of the variables) of 91.2 and 95.6% could be explained by the first two dimensions in **Figures 6A,B**, respectively, suggesting that the plots explained most of the variance quite well. Several key features can be observed from the plots: (i) all target genes were well separated in the plots (**Figure 6A**), confirmed by the Kruskal–Wallis ANOVA tests with *p*-values significantly less than 0.05 (**Table 4**), suggesting that the RT-qPCR and CA analysis was able to differentiate the independent gene-expression distribution patterns exhibited by each of the target genes (Theodorsson-Norheim, 1986; Shi et al., 2013; Qi et al., 2014); (ii) in the CA plot (**Figure 6A**), single cells at exponential phase all distributed in the left, while single cells at stationary phase all distributed in the right of the CA plots. In addition, single cells in the biofilm located in the upper of the CA plot, while single cells in the planktonic culture located in the bottom of the CA plot, indicating significant differences between single cells from four growth conditions and the distributions of four growth conditions in the CA plot were independent as also confirmed by the statistical analyses; (iii) in the CA plot based on the relationship between target genes and four growth conditions (**Figure 6B**), DVU0281 and DVU1340 genes were more close to the exponential phase in the biofilm, while DVU1397 was more close to the stationary phase in the biofilm, suggesting that they could be functionally related to the corresponding condition according to the CA theory (Greenacre, 1984; Yelland, 2010). Similarly, DVU3062 was far away from both BE and BS conditions in the plot, suggesting it might play little role in the formation and metabolism of the *D. vulgaris* biofilm.

**Table 4:** *P* values of Kruskal–Wallis tests at the 95% Confidence level.

Gene ID	*P*-value
DVU0281	5.156E-18
DVU0434	2.790E-7
DVU0588	1.729E-18
DVU1340	1.856E-21
DVU1397	1.673E-22
DVU2410	9.803E-12
DVU2571	0.016
DVU3062	2.862E-11

## Conclusion

In this study, we applied a single-cell RT-qPCR based approach to compare gene-expression levels of selected target genes in *D. vulgaris* grown in biofilm and planktonic cultures. While significant gene-expression heterogeneity was found for the selected genes in both the *D. vulgaris* biofilm and planktonic culture, the analysis showed that gene-expression heterogeneities of 7 out of 8 selected genes were clearly increased in single cells isolated from the *D. vulgaris* biofilm when compared with those from the planktonic culture, implying a possible linkage between gene-expression heterogeneity and structural and phenotypic complexities of the *D. vulgaris* biofilms. Interestingly, the analysis showed that compared to planktonic culture, expression levels of DVU1340 encoding ferric uptake repressor family transcriptional regulators and DVU1397 encoding the iron storage protein bacterioferritin were up-regulated for the *D. vulgaris* biofilms, while the expression level of DVU2571 encoding ferrous iron transport protein were down-regulated, suggesting their roles in maintaining the normal metabolism of the *D. vulgaris* biofilm under high concentration of iron. Finally, the results demonstrated that single-cell RT-qPCR analysis could be a valuable tool to reveal cell-level and micro-scale differences in the *D. vulgaris* biofilm, which could be applied in the future to understand molecular mechanism related to growth, maintenance and functions of various microbial biofilms.

## Author Contributions

ZQ performed experiments and data analysis, and wrote the manuscript. LC conceived, designed experiments and wrote the manuscript. WZ conceived and designed experiments, analyzed the data and wrote the manuscript.

## Conflict of Interest Statement

The authors declare that the research was conducted in the absence of any commercial or financial relationships that could be construed as a potential conflict of interest.

## Supplementary Material

FIGURE S1 **Standard curve to determine protein and carbohydrate levels for biofilm and planktonic cells of *D. vulgaris.* (A)** Standard curve of carbohydrate by using the anthrone-sulfuric acid colorimetry with sucrose as the standard. **(B)** Standard curve of protein by using the Bradford assay with bovine serum albumin as the standard. Correlation coefficients (square values – *R*^2^) and correlation equation are shown.

FIGURE S2 **Scanning electron microscope imaging of *D. vulgaris* biofilm grown on mild steel slides.** Image was taken on a Hitachi S-4800 SEM scanning electron microscope. Magnification is 10,000 × and th magnification bar (in micrometers) is 5 μm. Experimental procedures: slides were rinsed once with 50 mM PBS (PH 6.5) and then placed in a fixative overnight. The biofilms were washed with ddH_2_O, and then dried at the critical point with a CO_2_ critical point drier.

FIGURE S3 **Evaluation of three candidates of internal control genes for the biofilm and planktonic cultures.** C_T_ is the number of qPCR quantification cycle, the fractional cycle number where fluorescence increases above the threshold. Each set of 12 cells from biofilm and planktonic culture were used to evaluate the consistency of the internal control genes. The standard deviations (SD) and means of the C_T_ values were calculated using the OriginPro 8.0 software.

FIGURE S4 **Gene-expression histograms and heterogeneity boxplots of biological replication of DVU0281 under the exponential phase in both biofilm and planktonic culture.** BE and PE are abbreviations for exponential phase in the biofilm and the planktonic culture, respectively. **(A)** Gene-express histograms among biological replications of DVU0281 under BE and PE, respectively. **(B)** Gene-express histograms of DVU0281 in all 31 single cells under BE and PE, respectively. **(C)** Gene-expression heterogeneity boxplots of biological replication of DVU0281 under BE and PE, respectively.

FIGURE S5 **Frequency distribution histogram of gene-expression heterogeneities for biological replications of DVU0281 under the exponential phase in both biofilm and planktonic cultures**.
