# Supplementary material for: Comparison of Transcriptional Heterogeneity of Eight Genes between Batch Desulfovibrio vulgaris Biofilm and Planktonic Culture at a Single-Cell Level
Source: Front Microbiol. 2016 Apr 27;7:597. doi: 10.3389/fmicb.2016.00597 (PMC4847118; doi:10.3389/fmicb.2016.00597)
Supplement: FIGURE S1 — Standard curve to determine protein and carbohydrate levels for biofilm and planktonic cells of D. vulgaris. (A) Standard curve of carbohydrate by using the anthrone-sulfuric acid colorimetry with sucrose as the standard. (B) Standard curve of protein by using the Bradford assay with bovine serum albumin as the standard. Correlation coefficients (square values – R2) and correlation equation are shown. [file Image_1.PDF]

Suppl. Fig. 1

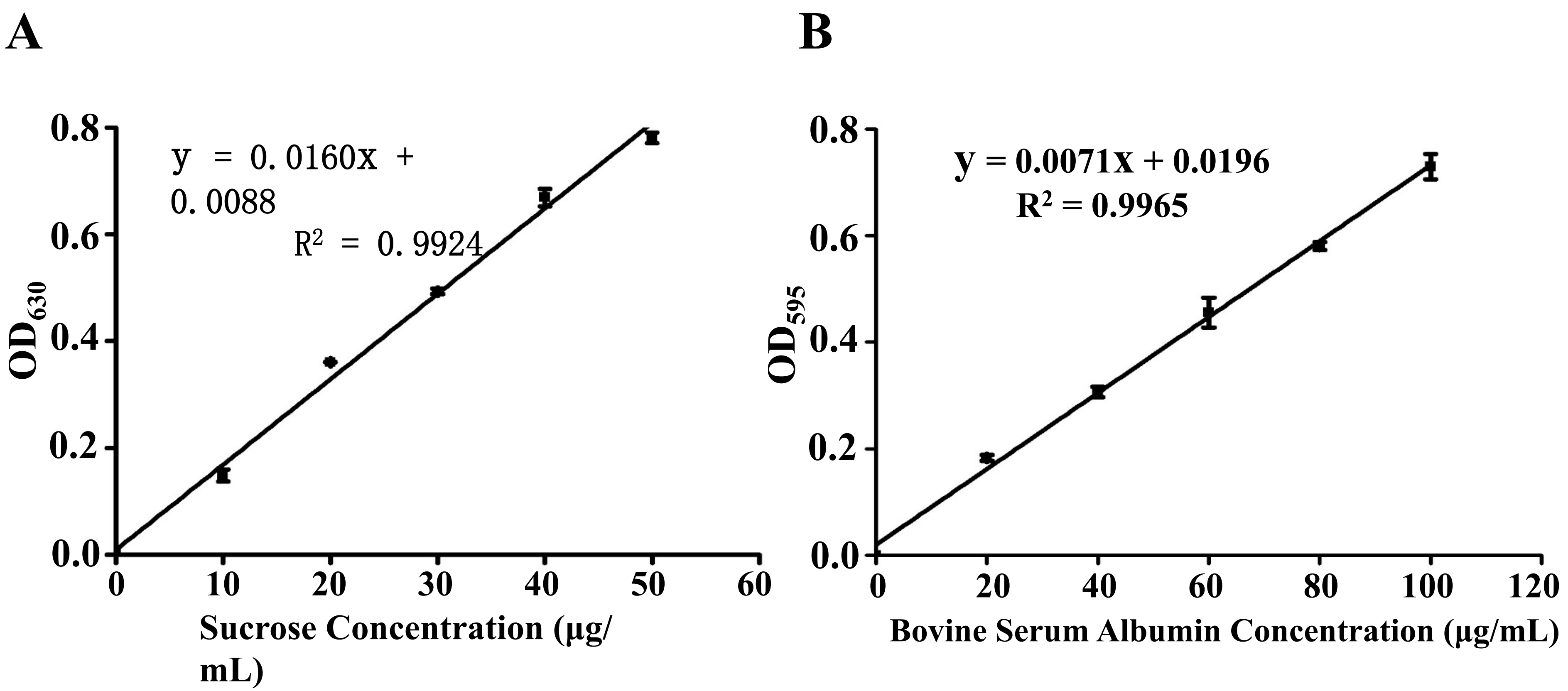
A
0.8
0.6
0.4
0.2
0.0
OD630
y = 0.0160x + 0.0088
R2 = 0.9924
0
10
20
30
40
50
60
Sucrose Concentration (µg/mL)
B
0.8
0.6
0.4
0.2
0.0
OD595
y = 0.0071x + 0.0196
R2 = 0.9965
0
20
40
60
80
100
120
Bovine Serum Albumin Concentration (µg/mL)

Suppl. Fig. 2

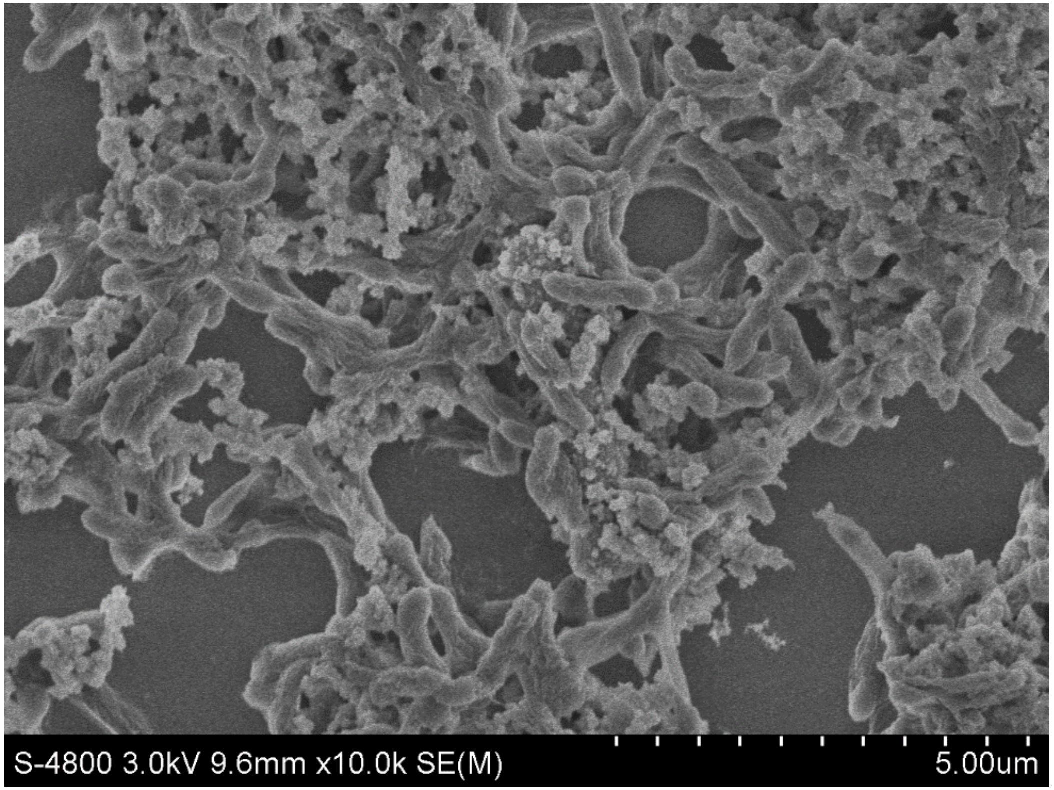

Suppl. Fig. 3

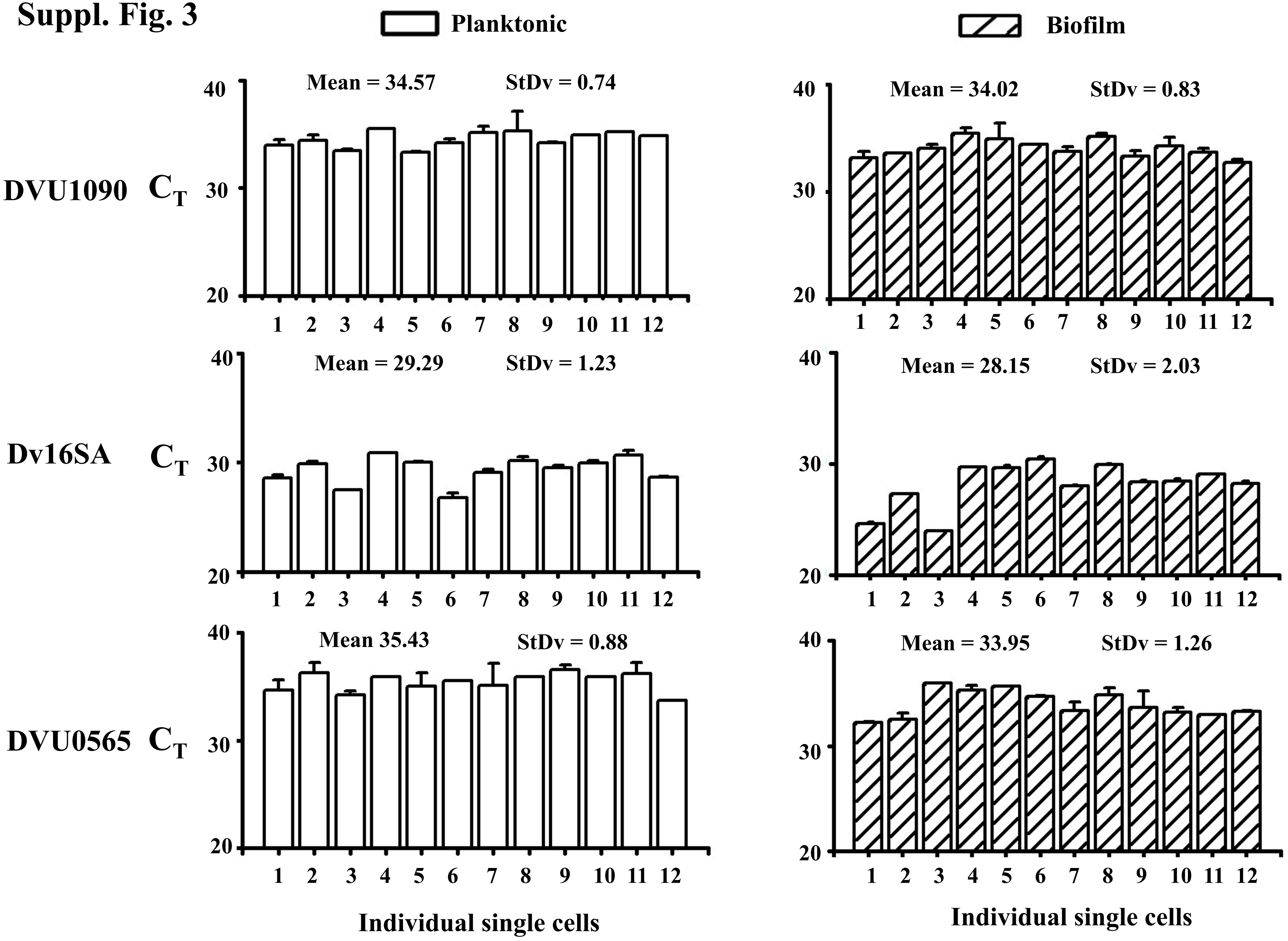

Planktonic
Biofilm
DVU1090
Dv16SA
DVU0565
C_T
Mean = 34.57
StDv = 0.74
Mean = 34.02
StDv = 0.83
Mean = 29.29
StDv = 1.23
Mean = 28.15
StDv = 2.03
Mean 35.43
StDv = 0.88
Mean = 33.95
StDv = 1.26
20
30
40
1 2 3 4 5 6 7 8 9 10 11 12
Individual single cells

Suppl. Fig. 4

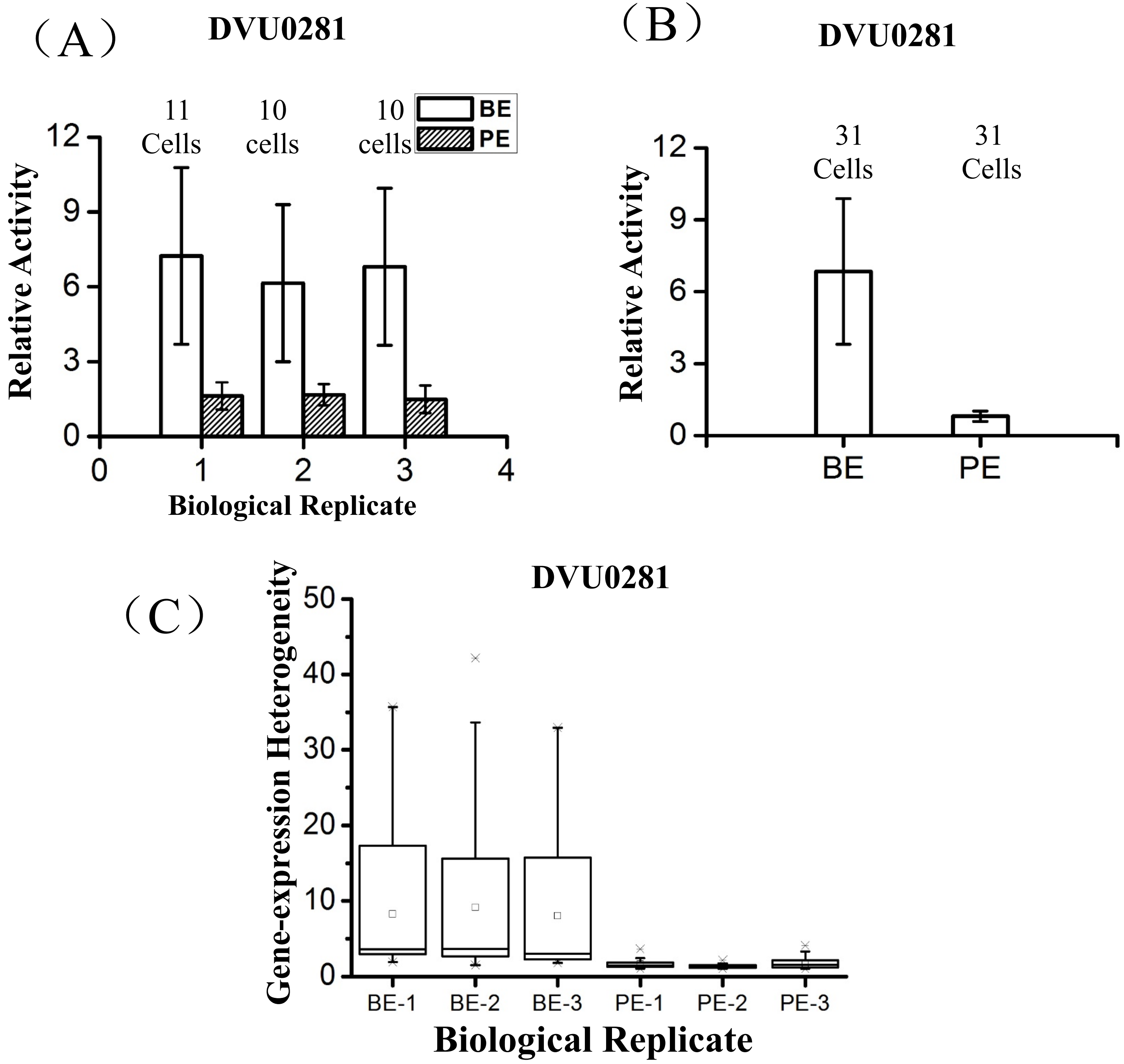
(A)
DVU0281
11 Cells
10 cells
10 cells
BE
PE
Relative Activity
0 3 6 9 12
0 1 2 3 4
Biological Replicate
(B)
DVU0281
31 Cells
31 Cells
Relative Activity
0 3 6 9 12
BE
PE
(C)
DVU0281
Gene-expression Heterogeneity
0 10 20 30 40 50
BE-1
BE-2
BE-3
PE-1
PE-2
PE-3
Biological Replicate

Suppl. Fig. 5

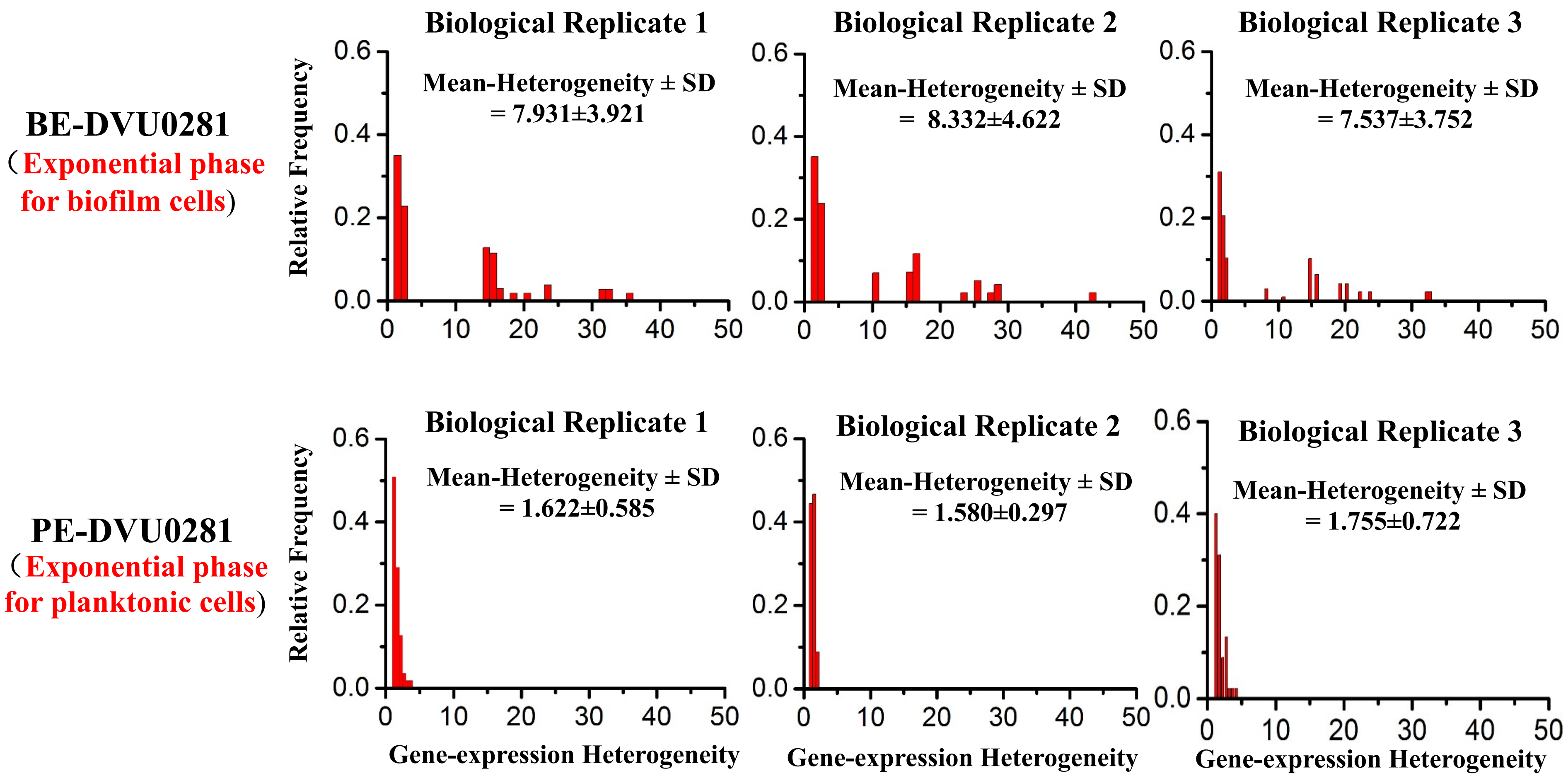
BE-DVU0281
（Exponential phase for biofilm cells）
PE-DVU0281
（Exponential phase for planktonic cells）
Biological Replicate 1
Biological Replicate 2
Biological Replicate 3
Mean-Heterogeneity ± SD = 7.931±3.921
Mean-Heterogeneity ± SD = 8.332±4.622
Mean-Heterogeneity ± SD = 7.537±3.752
Mean-Heterogeneity ± SD = 1.622±0.585
Mean-Heterogeneity ± SD = 1.580±0.297
Mean-Heterogeneity ± SD = 1.755±0.722
Relative Frequency
Gene-expression Heterogeneity
0.0
0.2
0.4
0.6
0
10
20
30
40
50
